# Navigation Using Sensory Substitution in Real and Virtual Mazes

**DOI:** 10.1371/journal.pone.0126307

**Published:** 2015-06-03

**Authors:** Daniel-Robert Chebat, Shachar Maidenbaum, Amir Amedi

**Affiliations:** 1 The Department of Medical Neurobiology, Institute for Medical Research Israel-Canada, Faculty of Medicine, Hebrew University of Jerusalem, Hadassah Ein-Kerem, Jerusalem, Israel; 2 The Edmond and Lily Safra Center for Brain Research, Hebrew University of Jerusalem, Hadassah Ein-Kerem, Jerusalem, Israel; 3 Department of Behavioral Sciences, Ariel University, Ariel, Israel; Emory University, UNITED STATES

## Abstract

Under certain specific conditions people who are blind have a perception of space that is equivalent to that of sighted individuals. However, in most cases their spatial perception is impaired. Is this simply due to their current lack of access to visual information or does the lack of visual information throughout development prevent the proper integration of the neural systems underlying spatial cognition? Sensory Substitution devices (SSDs) can transfer visual information via other senses and provide a unique tool to examine this question. We hypothesize that the use of our SSD (The EyeCane: a device that translates distance information into sounds and vibrations) can enable blind people to attain a similar performance level as the sighted in a spatial navigation task. We gave fifty-six participants training with the EyeCane. They navigated in real life-size mazes using the EyeCane SSD and in virtual renditions of the same mazes using a virtual-EyeCane. The participants were divided into four groups according to visual experience: congenitally blind, low vision & late blind, blindfolded sighted and sighted visual controls. We found that with the EyeCane participants made fewer errors in the maze, had fewer collisions, and completed the maze in less time on the last session compared to the first. By the third session, participants improved to the point where individual trials were no longer significantly different from the initial performance of the sighted visual group in terms of errors, time and collision.

## Introduction

There is a general consensus that the blind can navigate using their remaining senses [1–8]. In certain very specific conditions, when spatial information is matched between visual and non-visual cues, they are not impaired in their ability to represent space [6–11] while using tactile [12], or auditory maps [13].

However, in most everyday situations, visual information is not readily available through other senses and spatial information is not functionally matched by non-visual cues, which are typically scarce [14, 15]. Furthermore, there are several lines of research indicating that congenital blindness impairs spatial cognition [16–18]. For example, congenital blindness encourages the development of an egocentric frame of reference [19, 20], and impairs the precise localization of sounds [21]. There are also differences in the alignment of cortical and sub-cortical spatial maps [22, 23], and an inappropriate integration of the input from the non-visual sensory modalities in congenital blindness [24]. In addition to the massive reorganization of cortical [25], and sub-cortical structures [26], the hippocampus of the congenitally blind also undergoes volumetric changes [27, 28]. These profound neural and behavioral changes may indicate a potential inability to represent space properly, even if given the appropriate sensory-spatial information.

Are differences in the ability to navigate between blind and sighted people simply based on a lack of current visual information? Would it be possible to improve the performance of the blind in a navigation task to the point where it would be similar to the performance of the sighted? Or does congenital blindness prevent neural development that fundamentally affects navigation? Sensory Substitution Devices (SSDs) can potentially help address this question by conveying visual information non-invasively via auditory [29, 30] and tactile [31, 32] cues. These devices have been used successfully for a wide range of tasks including object identification [33, 34] and acuity [35, 36], obstacle avoidance [37] and others [38]. Here, we used the EyeCane minimalistic-SSD developed in our lab (see methods for expansion) [39] and the virtual-EyeCane that mimics it in virtual environments [40], which transforms distance to sound and vibration to address this question.

We hypothesized that relaying of distance information via the EyeCane and virtual EyeCane should enable blind people to perform in a navigation task equivalently to sighted participants. If despite the supplementation of the visuo-spatial information, the blind groups do not approach the performance of the sighted this may be due to underlying neural changes in spatial cognition in congenital blindness. In this latter case we expected to find differences between the congenitally blind and the other groups (late blind, low vision and sighted blindfolded) in terms of performance in the navigation tasks.

This is the first study to compare the performance of different types of blind individuals with that of sighted-visual control participants and sighted-blindfolded controls in both real life-size and virtual reality mazes using SSDs. In experiment 1 we used a bare room where participants could perceive the exit from their starting position with the use of the EyeCane, or visually in the case of the sighted-visual controls. Participants were asked to find the most direct route to the exit. We predicted that all the participants would behave similarly to the sighted-visual controls and take the most direct route to the exit. We tested whether even in the first session participants could perform as well as sighted-visual controls. In experiment 2, participants navigated a Hebb-Williams maze [41]. In this environment the exit is not perceptible from the starting position (through vision or EyeCane) and had multiple decision points and turns [42]. We tested whether after experience participants could perform as well as the sighted-visual controls.

We found that with the supplementation of the EyeCane, the performance of congenitally blind participants after training approached the initial performance of the sighted full vision controls. This may indicate that the previously reported problems are indeed due to a lack of current information and not to underlying inability to represent space in the congenitally blind.

## Materials and Methods

### Participants and Ethics

Thirty-six sighted healthy subjects participated in the study: twenty-three (15 women, range: 21–51 years, average: 28 years, mode: 24 years) were blindfolded, designated as the " sighted blindfolded" group, and thirteen sighted participants, designated as the "sighted visual" group performed the task using vision (8 women. range: 19–55 years, average: 28 years, mode: 21 years); twelve congenitally blind participants (range: 23–59 years, average: 36 years, mode: 23 years) all with documented total blindness from birth (except one participant before the age of 6 months), designated as the "congenitally blind" group. In addition, eight participants diagnosed with low vision or late acquired blindness (1 woman, range: 21–60 years, average: 40 years, mode: 21 years), designated as the "low vision & late blind group (LvLb)". Blindness was peripheral in all cases. The demographics of the blind participants are summarized in Table 1. All blind participants were adept white cane users, and had previously received orientation and mobility training. An additional 5 sighted participants took part in a drawing classification control experiment.

**Table 1:** 

*Name*	*Age*	*Sex*	*Group*	*Onset*	*Light Perception*	*Cause of Blindness*
DA	59	M	Congenitally Blind	birth	None	Retinopathy of Prematurity
MD	23	M	Congenitally Blind	birth	None	Congenital Glaucoma
UM	41	M	Congenitally Blind	6–7 mos	None	Retinopathy of Prematurity
OB	38	M	Congenitally Blind	birth	None	Retinopathy of Prematurity
SS	54	M	Congenitally Blind	birth	None	Retinopathy of Prematurity
OG	38	F	Congenitally Blind	birth	None	Anophtalmia
ED	33	M	Congenitally Blind	birth	None	Retinopathy of Prematurity
EH	30	F	Congenitally Blind	birth	Faint	Retinopathy of Prematurity
EN	30	F	Congenitally Blind	birth	None	Anophtalmia
MS	37	F	Congenitally Blind	birth	None	Anophtalmia
DH	35	F	Congenitally Blind	birth	None	Retinopathy of Prematurity
BJ	23	M	Congenitally Blind	birth	None	Microphtalmy
IB	33	F	Low Vision	birth	Faint	Glaucoma
MY	21	M	Low Vision	birth	Faint	Retinitis Pigmentosa
SA	21	M	Low Vision	2–3 mos	Faint	Retinitis Pigmentosa
SB	60	M	Low Vision	birth	Faint	Retinitis Pigmentosa
VG	60	M	Low Vision	birth	Faint	Craniosynostosis
MP	54	M	Late Blind	44 yrs	None	Diabetic Retinopathy
AS	27	M	Late Blind	15 yrs	Faint	Medical Accident
HA	49	M	Late Blind	43 yrs	None	Medical Accident

All fifty-six participants signed informed consent forms. The experiment was approved by the Hebrew University’s Ethics Committee and conducted in accordance with the 1964 Helsinki Declaration.

### The EyeCane

**Fig 1:**
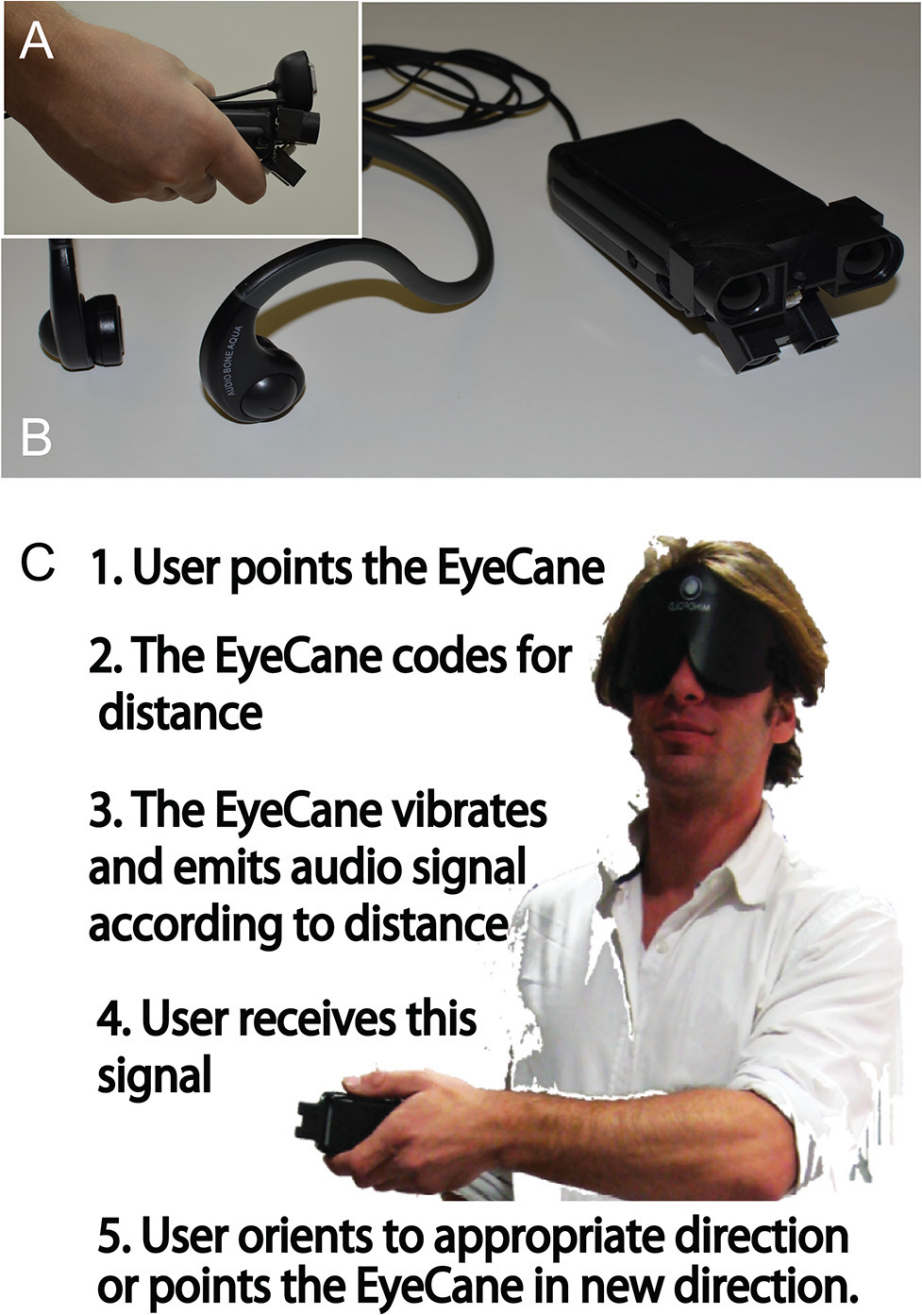
The EyeCane. A. A side view of the EyeCane and the IR sensors that capture the distance to the object it is pointed at. B. A top view of the EyeCane showing the headphones that transfer the distance information to the user into sound. A built-in vibrating motor also codes this distance information. C. The five steps in the sensory-motor loop and an image of the user pointing the device.

The EyeCane (see Fig 1A side view, and B top view) is a minimalistic-SSD developed by our team [39, 40, 43]. It has been used in other tasks such as distance estimation, obstacle detection and avoidance in virtual worlds [40], and in the real world [43]. A key feature of the EyeCane is its provision of real-time feedback (Fig 1C). We have previously demonstrated that new users can master its use within 5 minutes of training [43].

The EyeCane utilizes narrow-beam infrared sensors (<5deg) that are sensitive to distance. The range of the device is 5 meters. The EyeCane converts distance information into pulsating sounds and vibrations through the use of headphones and a built-in vibrating motor that can be felt on the palm of the hand (Fig 1A, 1B and 1C). A higher frequency rate of sounds and vibrations indicated a closer object [39, 40, 43]. Spatial information is perceived by physically sweeping the device to scan the environment, thus enabling the construction of a mental representation of the users' surroundings. By pointing the device at objects, the distance is translated into vibrations and auditory cues that inform the user of the distance to them (Fig 1). The EyeCane is lightweight, low-cost and updates in real-time (50Hz).

### The Real World Maze

**Fig 2:**
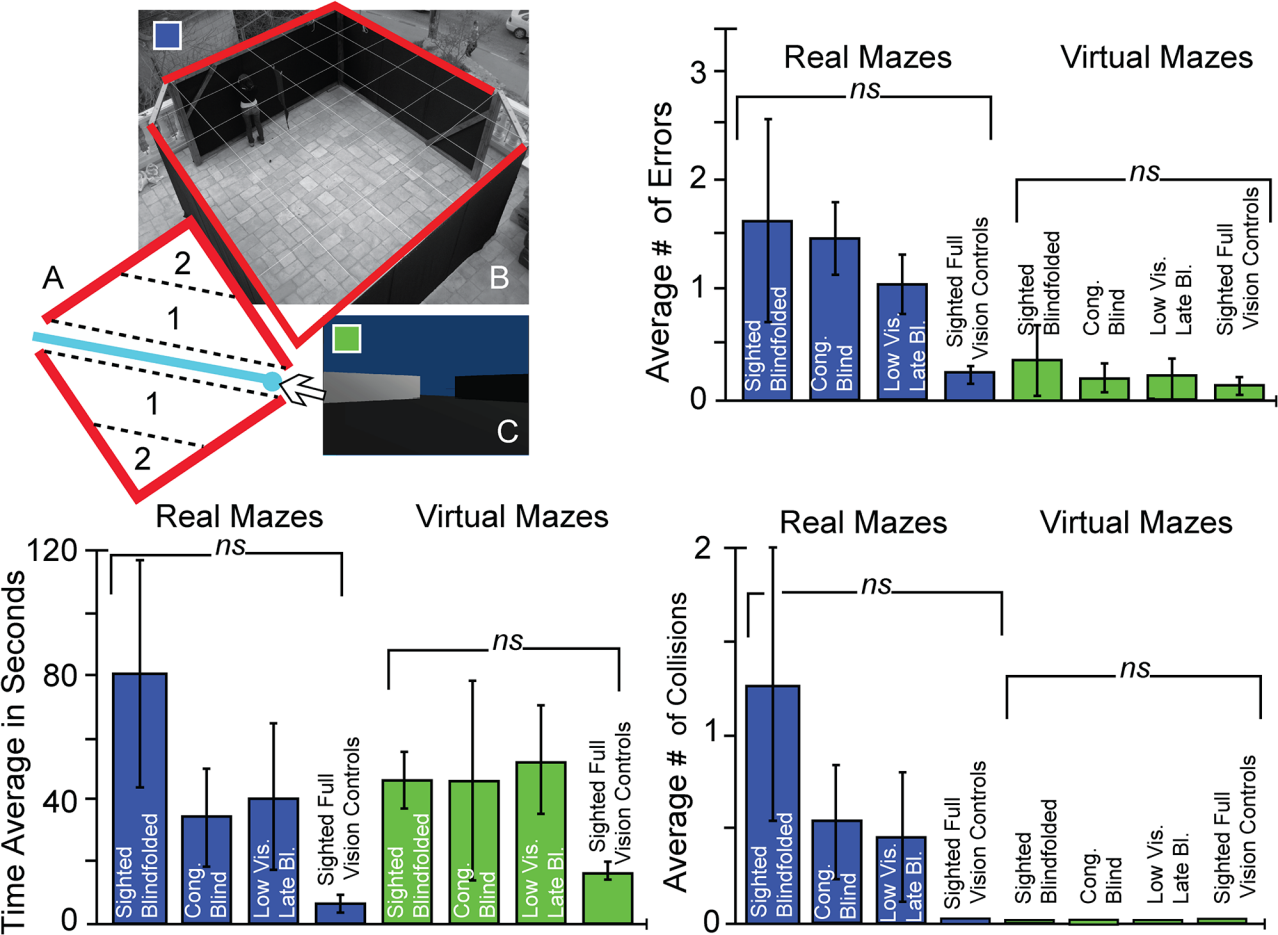
Experimental Setup 1 and Results. A. A diagram of the maze with the correct path in blue and the error zones delimited by the dotted lines. B. A top-down view of the real world maze. C. A first- person view of the virtual world maze. Bottom Left Panel: Time averages for all participants in the real world maze (blue) and the virtual maze (green). Top Right Panel: Error averages for all participants in the real world maze (blue) and the virtual maze (green). Bottom Right Panel: Collision averages for all participants in the real maze (blue) and the virtual maze (green). Asterisks indicate the level of significance. *p<0.05; **p<0.01; ***p<0.001.

**Fig 3:**
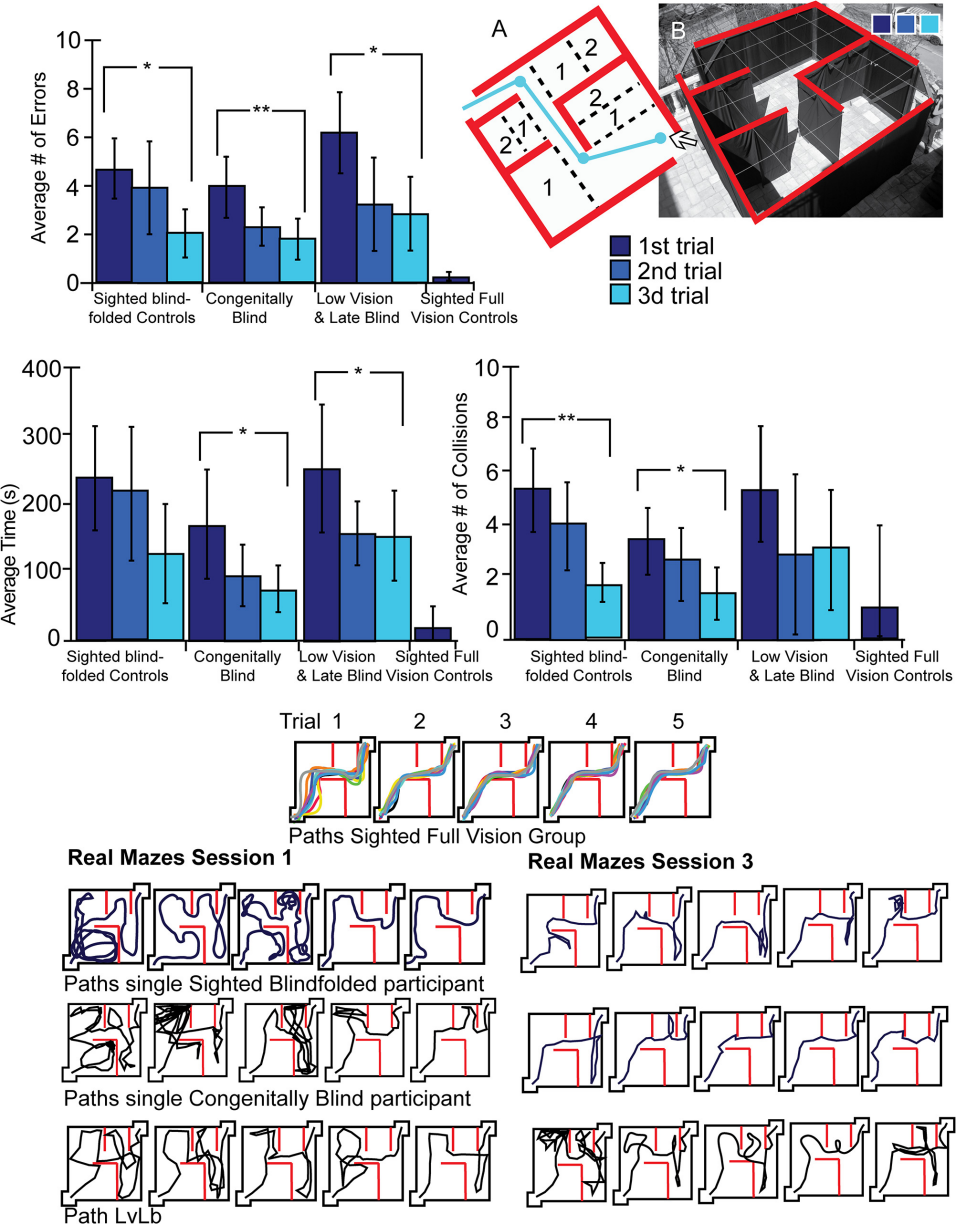
Real World Experimental Setup 2 and Results. A. A diagram of the maze with the correct path (in blue) and the error zones (dotted line). B. A top view of the real world maze. Top Left: Error averages for all participants in the real world maze. Dark blue indicates the performance on the first session, royal blue indicates the performance on the second session, and light blue on the third session in the real world mazes. Top Right: Collision averages for all participants in the real maze. Top Left: Time averages for all participants in the real world maze. Asterisks indicate the level of significance. *p<0.05; **p<0.01; ***p<0.001.Bottom Panels: Diagrams depicting the paths chosen by participants to complete the maze. Middle: All paths of the sighted full vision control participants. Left: Performance of participants on the first session in the real world maze. Right: Performance of participants on the third session in the real world maze.

**Fig 4:**
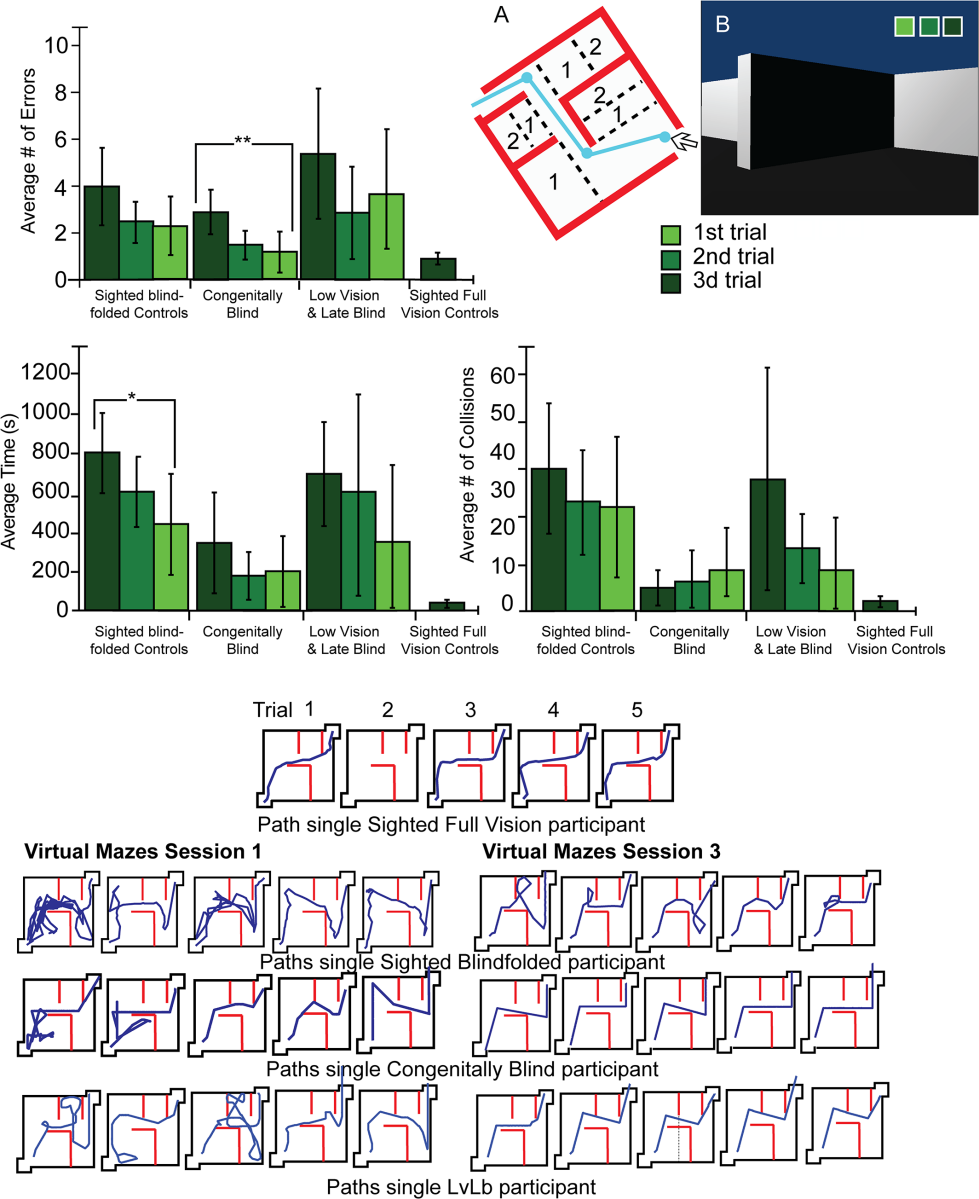
Virtual World Experimental Setup 2 and Results. A. A diagram of the maze with the correct path (in blue) and the error zones (dotted line). B. A first- person view of the virtual maze at the entrance. Top Left: Error averages for all participants in the virtual world maze. Dark green indicates the performance on the first session, forest green the performance on the second day of training, and light green the performance on the third day of training. Top Right: Collision averages for all participants in the virtual maze. Top Left: Time averages for all participants in the virtual maze (green). Asterisks indicate the level of significance. *p<0.05; **p<0.01; ***p<0.001. Bottom Panels: Diagrams depicting the paths chosen by participants to complete the maze. Middle: All paths of the sighted full vision control participants. Left: Performance of participants on the first session in the virtual world maze. Right: Performance of participants on the third session in the virtual maze.

Hebb-Williams mazes [41] are employed to test spatial perception and have been implemented in tests of spatial perceptual learning in a wide variety of species from mice [44] to monkeys [45], and even in a virtual rendition for humans [42]. The square maze used here measured 4.5 meters per side and was two meters high (for a total 20.25 square meters). There was an entrance in one corner and an exit in the opposite corner. Maze 1 was an empty room where the exit was on a straight line from the entrance (Fig 2A, 2B and 2C), and Maze 2 was more complex, comprising several turns and decision points (see Fig 3A, 3B and Fig 4A). Similar Hebb-Williams mazes have been classified in terms of difficulty for humans [42].

### The Virtual-EyeCane and Virtual World Maze

The virtual environments [40] were created with Blender 2.49, and Python 2.6.2. The location and orientation of the user’s avatar and Virtual-EyeCane were tracked at all times at a rate of 60 Hz (identical to the function rate of the virtual environment, thus covering any possible in-game activity) to enable re-creation and analysis of the participants’ errors, collisions and time. The environments have a graphical output on the screen (Figs 2C and 4B), which was used to track the participants’ progress during the experiment. The participants always experienced the environments in the 1^st^ person and not as a map overview. The virtual mazes were created to match the real world mazes in terms of structure (i.e. walls, corners and openings). Distances within the environment are set so that the proportions of a step compared to a “virtual meter” correlates to a real world step compared to a meter (i.e. the real world maze was 4.5m, so the virtual maze was set to 4.5 virtual meters, as was the scale of the avatar's size and motion; each step measured 0.5 virtual meters). The Virtual-EyeCane has previously been proven effective in increasing the accessibility of virtual environments, albeit far simpler ones, to the blind [40].

### Experimental Procedures

All the participants (except for the sighted full vision control group) were blindfolded, even the completely blind individuals, for purposes of homogeneity. The task was to find the fastest route to the exit while avoiding collisions (touching) with the walls. Participants stood at the entrance to the maze and held the EyeCane while wearing headphones that transmitted the distance information based on the EyeCane's cues. They were informed that a high frequency of sound meant a nearby wall, that the lower the frequency of sound, the further away the wall was, and that the absence of sound meant that the passage was clear. Participants were instructed to use the sounds of the EyeCane to scan the environment and build a mental image of the maze to find the shortest route to the exit. In the virtual version of the mazes, participants were seated comfortably in front of the computer, wearing headphones and received the distance information based on the same low/high rate of auditory cues. Participants navigated with the help of the arrow keys on the keyboard. The forward arrow key enabled a step forward, the right arrow key a turn to the right and the left arrow key a turn to the left.

In both the real and virtual mazes, an error was tabulated every time a participant deviated from the path and entered an error zone (Figs 2A, 3A and 4A). Collisions were counted as every time a participant touched or made any contact with a wall. If the participant continued to have contact with the wall, an additional collision was counted at each step. Time was measured from the moment the participants entered the maze until they exited it. Errors, time and collisions were noted by the experimenter during each trial in addition to the video recording in the real world and automatic logging in the virtual one, enabling repeated viewing as often as necessary to count the errors, collisions and time.

### Finding an exit in a simple maze

Participants searched for the exit in a bare, empty room five times in a row. The correct path was a direct route to the exit, without any turns or deviations (Fig 2A, 2B and 2C). After completing this real maze 5 times they were instructed to do the same task in the virtual mazes.

### Perceptual learning of a complex maze

In each of the three sessions, participants completed the Hebb-Williams maze five times in the real environment. Then, they were instructed to navigate the virtual maze. On the second and third session, the blindfolded participants returned and repeated the same sequence of five real maze and virtual maze trials five times. The correct path in the Hebb-Williams maze was more complex since it required several turns and decision points (Fig 3A). Each session lasted ninety minutes to two hours. The sighted-visual controls did the exact same task with the use of vision in one single block of five trials that lasted less than one hour.

**Fig 5:**
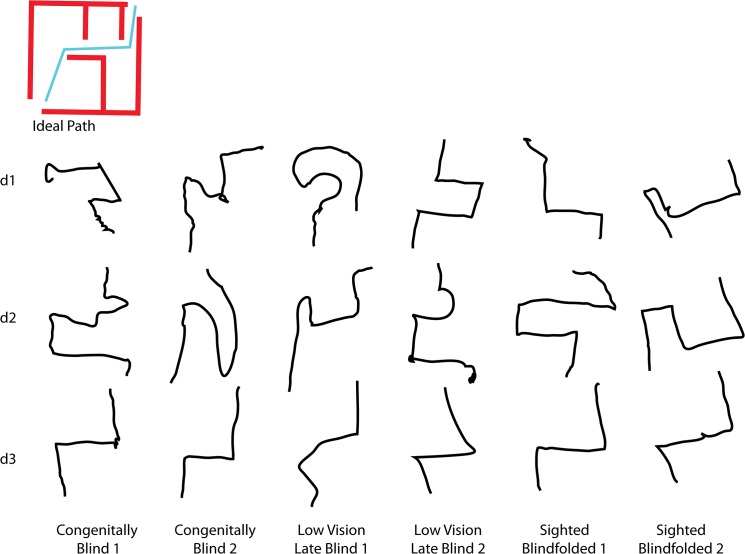
Actual drawings of the solution to the maze by participants. Examples of actual drawings of the paths by two participants from each blindfolded group (congenitally blind, LvLb, and the sighted blindfolded group) at the end of experimental sessions 1–3. Participants developed a more acurate mental representation of the spatial layout of the path to complete the maze over time.

At the end of each session, all the blindfolded participants were asked to make a pencil and paper drawing of the route to verify that they had encoded a cognitive map (Fig 5). These drawings were assessed subjectively by a control group of 5 independent assessors. The group of independent assessors received 16 random drawings and for each had to determine whether it was from the first or third day, the group, and rate it's quality as a solution to the maze from 1–5 (5 high).

### Statistical Analyses

We used the SPSS statistical package (IBM SPSS Statistics 20 Software) for the analyses; i.e., factor analyses, MANOVAs and ANOVAS and post hoc (LSD and Bonferroni) tests.

### Factor Analyses

Exploratory factor analyses were run on the five trials over the three days of testing, for each of the three measures of performance for the participants in both experiment 1 and experiment 2; that is, *Error*, *Time and Collision*. The factor analyses showed one factor for each measure (explaining respectively 62, 66 and 56% of the variance). All Cronbach’s alphas were higher than the recommended level required by Nunnally and Bernstein [46], that is .70 (α (*Error*) = .83; α (*Time*): = .86; α (*Collision*) = .79). We then created three composite variables for error, time and collision. These composite variables were used to compare performances between groups.

### Corrections for Multiple Comparisons

The procedure recommended by Saville [47] was followed. We examined the P values and confidence intervals of all comparisons made between the four groups of participants in all experimental conditions. The study involved 180 comparisons (i.e., 2 mazes x 6 pairs of subjects x 5 trials x 3 dependent variables). The probability that at least one of them was found significant was [1- (1-.05)180], that is almost 1. It was likely that 5% of the comparisons were significant [48], that is 9 comparisons .42 comparisons between pairs of groups showed significant differences, meaning that the significant results are likely not to be random.

### Low Vision and Late Blind Participants (LvLb)

Over both experiments, there were no significant difference between late blind and low vision participants on the three measures of *Error*, *Time* and *Collision* taken together (F(15, 91) = 0.95; p = 0.515), or separately (all F (1, 91)<. 1.39; p = 0.24). Therefore, the two groups of participants were merged and designated "Late blind and low vision (LvLb)".

## Results

### Finding an exit in a simple maze

All the participants were able to use the EyeCane and found the exit in less than 120 seconds in the real mazes and in less than 80 seconds in the virtual maze with the Virtual-EyeCane. The analyses of the real mazes revealed no significant differences in terms of errors (F(3,36) = 0.68; p = 0.57), time (F(3,36) = 2.58; p = 0.07) or collisions (F(3,36) = 2.06; p = 0.13) between groups. In the virtual maze, visual experience did not have an effect on the number of errors (F(3, 36) = 0.53; p = 0.66) or the amount of time (F(3,36) = 0.36; p = 0.79). There were no collisions for any of the participants in the simple virtual route (Fig 2).

### Perceptual learning of a Complex Maze

The next sections analyze the effects of visual experience on the three performance variables in both the real and virtual mazes in two different ways:

Comparing the performance of the groups before and after experience with the device: effect of learning (first session compared to the last session in the real and virtual mazes).

Differences between groups on the first and the last session in the real and virtual mazes, overall and then in terms of the different trials.

### Comparing performance before and after the EyeCane experience: Effect of Learning in the Real Maze

We tested the extent to which participants improved their performance from the first session to the last. In the real maze, all participants improved in terms of errors (Congenitally Blind: p<0.005; Sighted Blindfolded: p<0.031; LvLb: p<0.007) (Fig 3 and Table 2).

**Table 2:** Results Real Maze.

		*Congenitally Blind*	*Sighted Blindfolded*	*LvLb*
**Errors**	Session 1	3.9	4.5	6.1
	Session 3	1.8	2	2.8
***Probability between sessions***	*P* value	0.005	0.03	0.007
**Time (sec)**	Session 1	169 s	239.1 s	252 s
	Session 3	73.6 s	126.5 s	154 s
***Probability between sessions***	*P* value	0.043	*n*.*s*.	0.038
**Collisions**	Session 1	3.6	5.5	5.5
	Session 3	1.75	2.1	3.3
***Probability between sessions***	*P* value	0.047	0.003	*n*.*s*.

The congenitally blind improved on time (p<0.043), and collisions (p<0.047). The sighted-blindfolded controls had significantly fewer collisions (p<0.003) and the LvLb improved in terms of time (p<0.038) (Fig 3 top panels and Table 2).

The changes in navigation strategy and the drop in the number of errors were also visible in the typical paths taken by participants on the first (Fig 3 bottom left panels), compared to the last session (Fig 3 bottom right panels), reflecting a better understanding of the spatial layout of the maze.

We also verified that participants developed a cognitive map of the path to complete the maze. To do so, we asked all participants to draw the path from the starting point to the exit of the maze. Fig 5 shows examples of the drawings by two participants from each blindfolded group (congenitally blind, LvLb, and sighted-blindfolded controls). As shown, the drawings become more precise over time. On the first day of the experiment, they tended not to be very accurate, and often added a corner, or several turns, sometimes in the wrong direction. On the last day however, the drawn routes accurately represented the solution to the maze.

### Comparing performance before and after the EyeCane experience: Effect of Learning in the Virtual Maze

In the virtual maze, the congenitally blind significantly improved in terms of errors (p<0.006), the sighted-blindfolded significantly improved in terms of time (p<0.041), but the LvLb group did not improve significantly on any of the variables (all p’s>0.064) (Fig 4). Averages for the first session and last session for each group in the virtual mazes are given in Table 3 and in Fig 4 (top panels). Improvement in spatial grasp of the environment can be inferred from the typical paths taken by participants in the virtual world to complete the maze on the first (Fig 4 bottom left panels), compared to the last (Fig 4 bottom right panels) session of training.

**Table 3:** Results Virtual Maze.

		Congenitally Blind	Sighted Blindfolded	LvLb
**Errors**	Session 1	2.8	3.9	5.3
	Session 3	1.2	2.3	3.6
***Probability between sessions***	*P* value	0.006	*n*.*s*.	*n*.*s*.
**Time (sec)**	Session 1	346.8 s	803 s	695.9 s
	Session 3	204.3 s	441 s	354.7 s
***Probability between sessions***	*P* value	*n*.*s*.	0.041	*n*.*s*.
**Collisions**	Session 1	5.9	35.9	5.3
	Session 3	10.8	26.3	10.6
***Probability between sessions***	*P* value	*n*.*s*.	*n*.*s*.	*n*.*s*.

### Differences between groups on the first session in the Real Maze

Sighted-visuals made significantly fewer errors than all the non-visual groups (all p’s<0.001), took significantly less time (all p’s<0.015) and made fewer collisions than the sighted blindfolded group (all p’s<0.008) The sighted-visual group outperformed the congenitally blind group as well in terms of collisions on the first session, though not significantly (p = 0.083)(Fig 3 and Table 2).

### Differences between groups on the first session in the Virtual Maze

The sighted-visual group made fewer errors than the sighted-blindfolded group (p<0.003), LvLb (p<0.00), and outperformed the congenitally blind group, though not significantly (p = 0.066). In terms of time, the congenitally blind were not statistically different from the sighted-visual (p’s>0.40). The sighted outperformed the LvLb and the sighted blindfolded groups in terms of time (all p’s<0.00). In terms of collisions, there was no significant difference between the sighted-visual and the congenitally blind, and LvLb (all p’s>0.278)(Fig 4 and Table 3).

### Differences between groups on the third session in the Real Maze and in terms of individual trials

We compared the initial performance of the sighted-visual to the performance of the congenitally blind, LvLb and sighted-blindfolded on the third session. The sighted-visual made fewer errors than all non-visual groups (sighted blindfolded: p<0.09; LvLb: p<0.00), but the difference was not significant for the congenitally blind group (congenitally blind: p>0.125). The sighted-visual took significantly less time than sighted-blindfolded and LvLb (all p’s<0.005). Although the sighted-visual did outperform the congenitally blind, this difference was not significant (p = 0.15). There was no significant difference in terms of collisions for all groups (all p’s>0.093).

**Fig 6:**
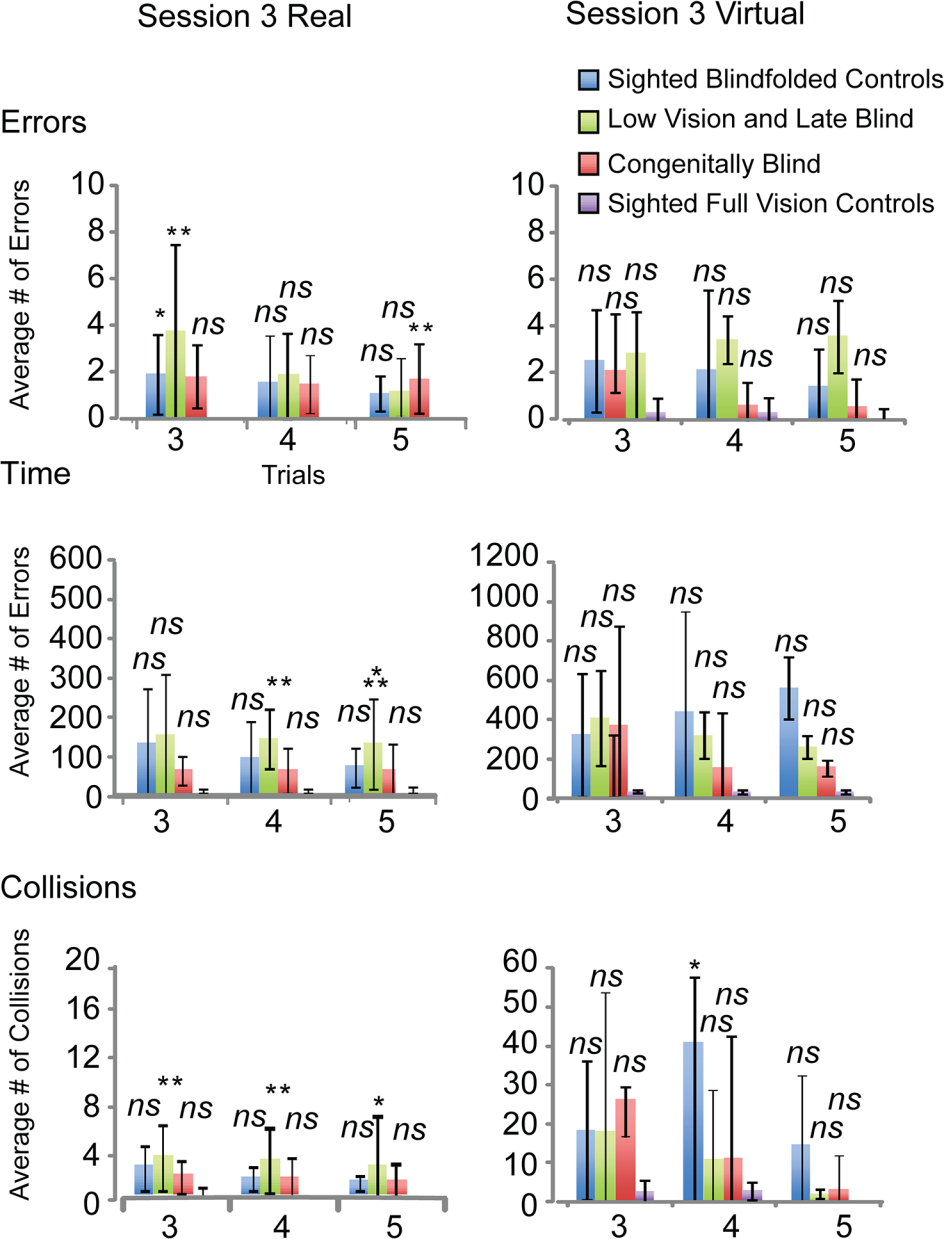
Real and Virtual performances on the last session. Differences between the initial performance of the sighted full vision controls and the other groups on the third session. All statistical comparisons were made with the initial performance of the sighted full vision controls. *p<0.05; **p<0.01; ***p<0.001.

The individual trials of the congenitally blind on the third session (Fig 6 right panels), were not statistically different from those of the sighted-visual on all trials in terms of errors (all p’s>0.29) except for the 5^th^ trial (p = 0.017). For the sighted-blindfolded, all trials were also not statistically different from the sighted-visual (all p’s > 0.132), except for the 3^rd^ trial (p = 0.03). The LvLb was not statistically different from the sighted-visual controls (all p’s >0.111), except for the 2^nd^ and 3^rd^ trials (p = 0.001, and p = 0.004 respectively) (Fig 6 top right panel). The LvLb took significantly more time than the sighted-visual on the 4^th^ and 5^th^ trials (p = 0.001, and p = 0.004 respectively). No other trials for all groups were significantly different from one another (all p’s > 0.05) (Fig 6 middle right panel). In terms of collisions, all congenitally blind (all p’s > 0.199) and sighted-blindfolded control (all p’s > 0.058) trials were similar to the sighted-visual. For LvLb, the first and second trials were not significantly different (both p’s = 1.00), but the 3^rd^, 4^th^, and 5^th^ trials were significantly different (p = 0.007, p = 0.002, and p = 0.052 respectively) (Fig 6 bottom right panel).

### Differences between groups on the third session in the Virtual Maze and in terms of individual trials

The sighted-visual made significantly fewer errors than the LvLb group (p<0.038) but not significantly fewer than the sighted-blindfolded (p = 0.21) or the congenitally blind (p = 0.79) (Fig 6 top left panel). In terms of time, the sighted-visual took significantly less time than the sighted-blindfolded (p<0.005) and the LvLb (p<0.049), but did not statistically outperform the congenitally blind (p = 0.24) (Fig 6 middle right panel). The sighted-visual made significantly fewer collisions than the sighted-blindfolded controls (p<0.008), but not statistically fewer than the congenitally blind (p = 0.340) or LvLb (p = 0.104) (Fig 6 bottom left panel Table 3).

For all trials in the virtual mazes, group performances were not significantly different in terms of errors (all p’s > 0.083). In terms of time, the congenitally blind were similar to the sighted-visual on all trials (all p’s > 0.277), as were the LvLb (all p’s > 0.295). For the sighted-blindfolded only the first and second trials were significantly different (p = 0.005, and p = 0.04 respectively); no other trials were significantly different from one another (all p’s > 0.07) (Fig 6). In terms of collisions the first and fourth trials of the sighted blindfolded participants were significantly different from the sighted full vision controls (p<0.05; and p<0.05 respectively).

### Drawing classification control

The group of independent assessors correctly recognized if the renditions of the paths drawn by participants (Fig 5) were from early or late session of the experiment (77.5%) indicating that there was a clear improvement. This is strengthened by their significant difference (p<2*10^-7) in rating (1–5 scale, 5 good) of the drawings from late mazes (4.2) vs. early mazes (2.5). Additionally, the independent assessors were not able to classify the routes by group better than chance (22.5%) indicating that the groups all perceived and drew with a similar level.

## Discussion

These results show that in the first experiment the three non-visual groups (congenitally blind, LvLb, sighted-blindfolded) performed as well as the sighted-visual group on a simple route task in finding the direct route to the exit.

The results from the second experiment show that all groups were able to learn to navigate in the Hebb-Williams maze with the EyeCane. During the experiment participants had to find their way and determine the correct route from the distance information delivered by the EyeCane. This led to improved spatial perception and the formation of a cognitive map, as witnessed by the improvement in navigation and the improvement in the drawings made by participants by the end of the experiment (Fig 5). All blindfolded groups (regardless of prior visual experience) could solve this complex maze with performances similar to the sighted-visual group.

These results suggest that differences in navigation between sighted and blind individuals may stem mainly from the lack of availability of visual information and are not due to underlying neural differences caused by visual deprivation.

From a practical rehabilitation aspect these results show that even the ability to sense the distance to a single point in space is enough to significantly improve navigation for blind users.

### Real vs. virtual

The findings demonstrate that all the participants could utilize the device well in both the real and virtual environments (Figs 2, 3 and 4). However we did not directly compare the two since the interaction with the environment on the two tasks was very different. Navigation in virtual mazes is constrained by movement speed and rotation angles and the lack of proprioceptive cues. Previous work in orientation and mobility training indicates that blind people can use virtual reality [49–51] and that the transfer of knowledge between real and virtual environments and vice versa is indeed possible [52–55], and can enable the use of virtual environments as a safe training platform for the real world. An important step in the future would be to explore this transfer of information between both types of environments in a practical setting.

### Modality independent representation

The present results support the current notion that the representation of space is amodal [56–60] (i.e., modality-independent) since an equivalent representation of space can be created from a SSD (Fig 5). The results suggest that mental spatial representations can be created using audition and vibration coding for distance, and that this representation can be abstracted from its modal source to represent space well enough to enable navigation (Figs 2, 3, 4, 5 and 6). The current results however do not indicate whether this representation includes independent maps for each.

### Extension of space

Previous research has demonstrated that multisensory space is extended by the use of the traditional white cane in blind cane users [61] and this extended touch can accurately depict a spatial location using a 2m probe [10]. Here, the EyeCane extends peri-personal space even further to 5m, with a similar effect that provides users with a distal sense. This significantly changes the sensory perception of the users' surroundings and contributed to the performances described here.

### Sensory Substitution devices—limits and horizons

When using SSDs the congenitally blind have better *visuo-tactile* acuity [35], and *visuo-auditory* acuity [36] than their blindfolded counterparts. They can use SSDs to recognize routes [60], detect and avoid obstacles [37–40, 43], recognize objects and shapes [33–36], and perceive depth [40, 43, 62, 63]. Despite these achievements SSD use for navigation outside laboratory settings remains extremely limited [39, 64].

Certain features of SSDs make them hard to use for navigation. Most SSDs are designed to transfer pictorial image-based information but are less suited to the rapid, real-time changes involved in everyday real-world navigation. They require great concentration because the interpretation of the information representing visual space is cognitively taxing. They are often cumbersome setups and require self-assembly. These limits and others [39] are likely to be drawbacks to their use for practical navigation. Recent technological advances however have mitigated many of these problems. The next generation of SSDs will doubtlessly include the tailoring of specific SSDs to different tasks. The EyeCane described here is one such minimalistic-SSD, in that it transfers only very specific and limited information, but does so in a suitable way for navigation. This can be seen in the key result of this work, that even distance information about a single point is enough to upgrade users' perception of their environment enough to allow for navigation in a complex environment.

Our results illustrate the potential of SSDs for spatial tasks and confirm other recent successful behavioral results using new SSDs, thus strengthening the potential of this approach. Future devices may benefit from these findings both in terms of a general backwind but also in terms of a more specific understanding of the utilization of minimal information and the importance of real-time active scanning over more complex substitutions.

## Conclusion

We showed that when using depth information for navigation all of the participants, regardless of visual experience, were able to navigate successfully through real and virtual, simple and complex environments, in a manner statistically similar to the sighted control participants. These results suggest that differences in navigation between sighted and blind individuals may stem from a lack of current visual information and are not due to underlying neural differences caused by visual deprivation. These results offer hope for future practical use of SSDs focused on conveying the missing perceptual information and utilizing this spatial representation.
